# Prevalence of Mild Autonomous Cortisol Secretion Among Patients With Adrenal Incidentaloma and Its Association With Metabolic Abnormalities: A Hospital-Based, Analytical, Cross-Sectional Study

**DOI:** 10.7759/cureus.116264

**Published:** 2026-09-15

**Authors:** Wardah Ikram

**Affiliations:** 1 Internal Medicine, Allama Iqbal Medical College, Jinnah Hospital Lahore, Lahore, PAK; 2 Endocrinology and Diabetes, National Health Service (NHS), London, GBR

**Keywords:** adrenal incidentaloma, diabetes mellitus, hypercortisolism, hypertension, metabolic syndrome, obesity

## Abstract

Background

Mild autonomous cortisol secretion (MACS) is a common hormonal abnormality among patients with adrenal incidentaloma and has been associated with cardiometabolic abnormalities. However, evidence regarding its prevalence and metabolic associations in Pakistani populations remains limited. This study aimed to determine the prevalence of MACS among patients with adrenal incidentaloma and evaluate its association with metabolic abnormalities.

Methodology

A hospital-based, analytical, cross-sectional study was conducted among 380 adults with adrenal incidentaloma at Jinnah Hospital, Lahore, affiliated with Allama Iqbal Medical College, Pakistan. Participants underwent clinical, anthropometric, biochemical, hormonal, and radiological assessment. MACS was evaluated using the 1-mg overnight dexamethasone suppression test, with assessment of morning adrenocorticotropic hormone (ACTH) and dehydroepiandrosterone sulfate to support ACTH-independent cortisol secretion. Metabolic abnormalities, including obesity, central obesity, hypertension, diabetes mellitus, dyslipidemia, and metabolic syndrome, were assessed. Multivariable logistic regression was performed to evaluate independent associations after adjustment for relevant demographic, lifestyle, clinical, medication-related, and radiological covariates.

Results

MACS was identified in 95 (25.0%) participants. Compared with patients without MACS, those with MACS had higher frequencies of obesity, central obesity, hypertension, diabetes mellitus, dyslipidemia, and metabolic syndrome, as well as a greater burden of multiple metabolic abnormalities. After multivariable adjustment, MACS was independently associated with obesity (adjusted odds ratio (aOR) = 1.85, 95% confidence interval (CI) = 1.09-3.14; p = 0.022), diabetes mellitus (aOR = 1.68, 95% CI = 1.01-2.80; p = 0.046), metabolic syndrome (aOR = 1.82, 95% CI = 1.11-2.98; p = 0.017), and the presence of ≥2 metabolic abnormalities (aOR = 1.92, 95% CI = 1.17-3.15; p = 0.010). Larger adrenal lesion size was independently associated with MACS (aOR = 1.38 per cm, 95% CI = 1.14-1.67; p = 0.001).

Conclusions

MACS was present in one-quarter of patients with adrenal incidentaloma and was associated with a greater cardiometabolic burden. These findings highlight the potential importance of assessing cortisol secretion and metabolic risk in patients with adrenal incidentaloma, while prospective multicenter studies are needed to clarify the clinical significance and temporal relationships of these associations.

## Introduction

Adrenal incidentalomas are adrenal masses discovered during imaging performed for clinical conditions unrelated to suspected adrenal disease. With the widespread use of computed tomography (CT) and magnetic resonance imaging (MRI), these lesions are increasingly recognized in routine clinical practice and are estimated to occur in approximately 1-7% of adults undergoing cross-sectional abdominal imaging. Most adrenal incidentalomas are benign and non-functioning; however, a clinically important proportion demonstrate autonomous hormone secretion, particularly cortisol excess [1]. Although overt adrenal Cushing syndrome is relatively uncommon, mild autonomous cortisol secretion (MACS) represents a more frequent biochemical phenotype characterized by autonomous cortisol production without the classical clinical manifestations of overt Cushing syndrome. The 2023 European Society of Endocrinology/European Network for the Study of Adrenal Tumors (ESE/ENSAT) guideline recommends a 1-mg overnight dexamethasone suppression test (DST) for patients with adrenal incidentaloma and considers a post-DST serum cortisol concentration >50 nmol/L (>1.8 µg/dL), in the absence of overt Cushingoid features, consistent with MACS [2,3].

The clinical importance of MACS has increased substantially because even relatively modest and clinically unapparent cortisol excess may be associated with adverse cardiometabolic outcomes. Chronic exposure to excess cortisol can promote visceral adiposity, insulin resistance, impaired glucose metabolism, hypertension, dyslipidemia, and other components of metabolic syndrome. An open-access systematic review reported that patients with cortisol-secreting adrenal incidentalomas had higher frequencies of hypertension, impaired glucose metabolism, dyslipidemia, and increased body mass index (BMI) than patients with non-functioning adrenal incidentalomas (NFAIs) [4]. More recent evidence has further supported these associations, with a systematic review and meta-analysis of more than 17,000 patients reporting higher risks of diabetes, hypertension, and dyslipidemia among patients with MACS than among those with NFAIs. The same analysis also found increased all-cause mortality, although the observational nature and heterogeneity of the available studies limit causal interpretation [5].

Despite growing recognition of MACS, its reported prevalence varies according to diagnostic criteria, cortisol thresholds, population characteristics, and testing strategies. A systematic review and meta-analysis reported an overall prevalence of autonomous or possible autonomous cortisol secretion of 11.7%, with a pooled prevalence of approximately 8.3% in Asian populations [6]. This geographical variation is particularly relevant because most available evidence has originated from European, North American, and East Asian populations, while evidence from South Asia remains comparatively limited. Differences in terminology and diagnostic thresholds across studies also make direct comparison between populations difficult. Use of contemporary MACS criteria and standardized DST-based assessment may therefore provide more comparable epidemiological data.

The Pakistani context makes this issue particularly important. Pakistan has a substantial and increasing burden of metabolic disease. A systematic review and meta-analysis of apparently healthy Pakistani adults estimated the pooled prevalence of metabolic syndrome at 28.8%, with central obesity, elevated triglycerides, and low high-density lipoprotein cholesterol (HDL-C) representing particularly common components [7]. Similarly, pooled estimates from Pakistani studies have reported a prevalence of diabetes of approximately 13.7-14.6% among adults [8,9]. In this setting, identifying potentially relevant endocrine contributors to metabolic abnormalities may have important clinical implications, particularly among patients with adrenal incidentaloma.

However, there remains a research gap in Pakistan, particularly in Lahore, regarding the prevalence and metabolic correlates of MACS among patients with adrenal incidentaloma. Although the increasing use of abdominal imaging has led to more frequent detection of adrenal incidentalomas, locally generated evidence on MACS remains limited. Furthermore, it is unclear whether MACS is independently associated with metabolic abnormalities after accounting for relevant demographic, clinical, lifestyle, medication-related, renal, and adrenal lesion characteristics. International evidence may not be directly generalizable to the Pakistani population because of differences in genetic, nutritional, socioeconomic, and healthcare factors. This study aimed to determine the prevalence of MACS among adults with adrenal incidentaloma and to evaluate its association with metabolic abnormalities, including obesity, hypertension, diabetes mellitus, dyslipidemia, and metabolic syndrome, after adjustment for relevant demographic, clinical, lifestyle, medication-related, renal, and radiological factors.

## Materials and methods

Study design and rationale

This hospital-based, analytical, cross-sectional study was conducted among adult patients with adrenal incidentaloma to determine the prevalence of MACS and evaluate its association with metabolic abnormalities. Participants were classified into MACS and NFAI groups according to predefined biochemical criteria. The study evaluated the relationships of MACS with obesity, hypertension, diabetes mellitus, dyslipidemia, metabolic syndrome, and other cardiometabolic abnormalities using standardized clinical, biochemical, hormonal, and radiological assessments.

Study setting

The study was conducted at Jinnah Hospital, Lahore, affiliated with Allama Iqbal Medical College, Punjab, Pakistan. Jinnah Hospital is a tertiary-care teaching hospital providing specialized medical, endocrine, surgical, urological, radiological, and laboratory services and serving patients from Lahore and surrounding districts. Patients with adrenal incidentalomas were identified through relevant clinical departments, including endocrinology, medicine, general surgery, urology, and radiology, when an adrenal lesion was detected incidentally during imaging performed for a clinical indication unrelated to suspected adrenal hormone excess. A standardized study protocol (Appendices) was used for participant selection, clinical and anthropometric assessment, blood pressure measurement, biochemical and hormonal investigations, imaging evaluation, and data recording. Because all participants were recruited from the same institution, the hospital site was constant across the study population and was not included as a covariate in the multivariable analyses.

Study duration

The study was conducted from August 2024 to July 2025 at Allama Iqbal Medical College, Lahore, Punjab, Pakistan, following approval from the relevant institutional ethics committee. Participant recruitment, clinical assessment, biochemical and hormonal investigations, radiological assessment, data verification, and statistical analysis were performed according to the approved study protocol during the study period.

Study population

The study population consisted of adults aged 18 years or older who had an adrenal incidentaloma identified during imaging performed for a clinical indication unrelated to suspected adrenal hormone excess. Adrenal lesions could initially have been detected through abdominal ultrasonography, CT, MRI, or another clinically indicated imaging modality. Available imaging was subsequently reviewed to characterize the adrenal lesion and assess its radiological features.

Participants were considered eligible when an incidental adrenaloma was documented, and a complete endocrine assessment required for classification of cortisol secretion was available. Patients with common metabolic conditions such as obesity, hypertension, diabetes mellitus, dyslipidemia, chronic kidney disease, or cardiovascular disease were not excluded because these conditions constituted important outcomes of the study.

Sample size calculation

The sample size was calculated using the single-population proportion formula because the primary objective was to estimate the prevalence of MACS among patients with adrenal incidentaloma. Previous international literature has reported substantial variation in the prevalence of autonomous cortisol secretion among patients with adrenal incidentaloma, generally ranging from approximately 15% to 30% depending on the diagnostic criteria and population studied [10-13]. An anticipated prevalence of 30% was therefore used for the present study to provide a conservative sample size for estimating the prevalence of MACS.

The sample size was calculated using a 95% confidence level and 5% absolute precision according to the following equation: $n=\frac{Z_{1-\alpha/2}^{2}p(1-p)}{d^{2}}$. n represented the minimum required sample size, Z1−α/2​ represented the standard normal deviate corresponding to the selected confidence level, p represented the anticipated prevalence of MACS, and d represented the desired absolute precision. Using Z1−α/2 ​= 1.96, p = 0.30, and d = 0.05, the calculation was:

$n=\frac{(1.96)^2(0.30)(1-0.30)}{(0.05)^2}$.

$n=\frac{3.8416\times0.30\times0.70}{0.0025}$

$n=\frac{0.806736}{0.0025}=322.69$

Thus, the minimum required sample size was approximately 323 participants. To compensate for incomplete hormonal testing, inadequate dexamethasone exposure, inability to complete confirmatory endocrine assessment, withdrawal of consent, missing biochemical measurements, or exclusion after identification of overt Cushing syndrome or another functional adrenal disorder, an additional 15% was added. Therefore, the final target sample size was 380 participants with adrenal incidentaloma.

Sampling technique

Eligible participants were recruited using consecutive sampling. Every adult patient who fulfilled the predefined eligibility criteria during the study period was considered for enrollment until the required sample size was achieved. Consecutive recruitment was selected to minimize investigator-driven selection and to avoid preferential recruitment according to age, sex, obesity, metabolic status, or cortisol concentration.

Recruitment was performed at Allama Iqbal Medical College, Lahore. The study site was recorded for each participant; because all participants were recruited at Allama Iqbal Medical College, Lahore, the recruitment site was not included as a variable in the multivariable analysis.

Inclusion criteria

Adults aged 18 years or older were included if they had a documented adrenal incidentaloma identified during imaging performed for a clinical indication unrelated to suspected adrenal hormone excess. Participants were included when clinical assessment and the complete hormonal evaluation specified in the study protocol, including assessment of post-DST cortisol, morning adrenocorticotropic hormone (ACTH), and dehydroepiandrosterone sulfate (DHEA-S), could be completed, and when they provided written informed consent to participate in the study.

Exclusion criteria

Patients with established overt Cushing syndrome were excluded from the primary MACS analysis. Patients with previously diagnosed functional adrenal disorders requiring immediate disease-specific management, confirmed pheochromocytoma, confirmed adrenocortical carcinoma, adrenal metastasis, or another clearly non-incidental adrenal lesion were also excluded.

Patients receiving systemic glucocorticoid therapy capable of interfering with hypothalamic-pituitary-adrenal axis assessment were excluded unless an appropriate clinical washout period could safely be achieved. Patients with pregnancy, severe acute systemic illness likely to interfere substantially with cortisol assessment, inability to complete the required endocrine assessment, or missing essential hormonal measurements were also excluded from the final analysis.

Participant recruitment and consent

Potentially eligible participants were identified through relevant clinical departments and radiology services. The study objectives, procedures, potential benefits and risks, confidentiality arrangements, and voluntary nature of participation were explained to eligible individuals. Written informed consent was obtained before enrollment and before study-specific assessments were performed. Each participant was assigned a unique study identification number. Names and other directly identifying information were not used in the analytical database.

Clinical assessment for overt Cushing syndrome

All participants underwent clinical assessment for features suggestive of overt Cushing syndrome before classification as MACS. Facial plethora, easy bruising, proximal muscle weakness, wide violaceous striae, unexplained or progressive weight gain, osteoporosis or fragility fractures, characteristic Cushingoid appearance, menstrual irregularity, and hirsutism were documented where applicable.

Participants with a clinical phenotype suggestive of overt Cushing syndrome were not classified as having MACS and were excluded from the primary MACS analysis. MACS was therefore restricted to participants without clinically overt Cushing syndrome who demonstrated biochemical evidence of autonomous cortisol secretion.

Anthropometric assessment

Body weight was measured using a calibrated digital weighing scale with participants wearing light clothing and no shoes. Height was measured using a calibrated stadiometer. Body mass index (BMI) was calculated using: $BMI=Weight (kg)Height (m)2BMI=\frac{\text{Weight (kg)}}{\text{Height (m)}^2}$. BMI was recorded both as a continuous variable and according to the predefined BMI categories used in the study. Waist circumference was measured using a non-stretchable measuring tape at the standardized anatomical site at the end of normal expiration. Two measurements were obtained, and their mean was recorded. Hip circumference was measured where required, and waist-to-hip ratio was calculated.

Blood pressure assessment

Blood pressure was measured using a validated automated sphygmomanometer after the participant had remained seated quietly for at least five minutes. An appropriately sized cuff was used according to arm circumference. Two blood pressure measurements were obtained at an interval of approximately one to two minutes. If the readings differed substantially, an additional measurement was obtained. The mean of the appropriate readings was used for analysis.

Hypertension was defined using the predefined diagnostic criteria specified in the study protocol and included participants with an established diagnosis and/or current antihypertensive treatment. The exact blood pressure threshold used for newly identified hypertension was applied consistently throughout the study.

Adrenal imaging assessment

All available adrenal imaging was reviewed using the radiology report and, where feasible, direct review of the original images by a qualified radiologist. The imaging modality was recorded as CT, MRI, ultrasonography, or another modality. The adrenal lesion was characterized according to laterality, number, maximum diameter, morphology, homogeneity, unenhanced CT attenuation in Hounsfield units (HU), contrast enhancement, absolute washout, relative washout where available, calcification, necrosis, hemorrhage, local invasion, lymphadenopathy, and other radiological features suggestive of malignancy.

The maximum lesion diameter was recorded in centimeters as a continuous variable. Lesion laterality was classified as right, left, or bilateral. The recruitment database retained the raw imaging measurements whenever available rather than recording only categorical radiological classifications.

Biochemical assessment

After an appropriate overnight fasting period, venous blood samples were collected for fasting plasma glucose, HbA1c, total cholesterol, triglycerides, HDL-C, low-density lipoprotein cholesterol (LDL-C), serum creatinine, estimated glomerular filtration rate, serum sodium, serum potassium, and other clinically relevant biochemical parameters.

All biochemical investigations were performed using the standard laboratory procedures of Allama Iqbal Medical College, Lahore. Laboratory values were recorded directly from laboratory reports rather than estimated from participant recall.

Diabetes mellitus, prediabetes, dyslipidemia, obesity, and central obesity were classified using the predefined diagnostic criteria specified in the study protocol.

Hormonal evaluation

A standardized hormonal assessment was performed in all 380 participants to determine cortisol autonomy. Specifically, all participants underwent assessment of post-1-mg overnight dexamethasone suppression cortisol, morning plasma ACTH, and serum DHEA-S. This ensured that ACTH and DHEA-S availability was not restricted only to participants with an abnormal DST and allowed consistent assessment of biochemical features supporting ACTH-independent cortisol secretion across the study population.

The primary screening test for cortisol autonomy was the 1-mg overnight DST. Dexamethasone 1 mg was administered orally at approximately 11:00 PM, followed by collection of a serum cortisol sample at approximately 8:00 AM the following morning. The post-DST serum cortisol concentration was recorded as a continuous variable.

A post-DST serum cortisol concentration of ≤50 nmol/L (≤1.8 µg/dL) was considered adequately suppressed, whereas a concentration >50 nmol/L (>1.8 µg/dL) was considered abnormal and compatible with MACS in the absence of overt Cushing syndrome. Morning ACTH and DHEA-S results were used as supportive biochemical evidence of ACTH-independent cortisol secretion and were interpreted in conjunction with the clinical findings and DST result.

Morning ACTH was categorized according to the laboratory reference range, with low or suppressed ACTH supporting ACTH-independent cortisol secretion. DHEA-S was recorded as a continuous variable and categorized according to the laboratory reference range where appropriate.

Because ACTH and DHEA-S were measured in all participants, their availability was not a source of differential endocrine assessment between the MACS and NFAI groups. Where the DST result was considered inconsistent with the clinical presentation, or where inadequate dexamethasone exposure or another potential source of a false-positive result was suspected, the DST was repeated where clinically appropriate.

Definition of mild autonomous cortisol secretion

MACS was operationally defined as an adrenal incidentaloma in a participant without clinically overt Cushing syndrome, a post-1-mg overnight DST serum cortisol concentration >50 nmol/L (>1.8 µg/dL), and biochemical evidence supporting ACTH-independent cortisol secretion, with no alternative explanation for abnormal cortisol suppression.

All 95 participants classified as having MACS were required to fulfill the prespecified MACS classification criteria. ACTH and DHEA-S measurements were used to support assessment of ACTH independence rather than serving as isolated diagnostic criteria. Participants with a post-DST cortisol concentration ≤50 nmol/L and no other evidence of cortisol autonomy were classified as having NFAI. Participants with clear clinical and biochemical evidence of overt Cushing syndrome were excluded from the primary MACS versus NFAI comparison.

Assessment of other adrenal hormonal disorders

Participants were assessed for evidence of other adrenal hormonal disorders where clinically indicated. Biochemical assessment for pheochromocytoma included plasma free metanephrines and/or urinary fractionated metanephrines according to clinical indication and institutional availability. In participants with hypertension, particularly when associated with hypokalemia or other clinical suspicion of primary aldosteronism, aldosterone and renin measurements were obtained where clinically appropriate, and the aldosterone-renin ratio was calculated. Additional adrenal hormonal investigations were performed when indicated by clinical or radiological findings. Participants with confirmed functional adrenal tumors other than MACS were not included in the primary MACS prevalence analysis.

Assessment of metabolic abnormalities

The principal metabolic outcomes included obesity, central obesity, hypertension, diabetes mellitus, prediabetes, dyslipidemia, and metabolic syndrome. Cardiovascular disease and other relevant cardiometabolic conditions were also recorded. Obesity was assessed using BMI, while central obesity was assessed using waist circumference according to the predefined study criteria. Hypertension was assessed using documented clinical diagnosis, antihypertensive treatment, and blood pressure measurements. Diabetes mellitus and prediabetes were determined from clinical history, medication use, fasting glucose, and HbA1c. Dyslipidemia was assessed using lipid profile results and relevant medication history. Metabolic syndrome was defined according to the prespecified diagnostic criteria used in the study, with individual components recorded separately. The total number of metabolic abnormalities present in each participant was also calculated to assess overall cardiometabolic burden.

Potential confounding variables and covariate selection

Potential confounding variables were identified before the final statistical analysis. These included age, sex, BMI or waist circumference, smoking status, physical activity, diabetes mellitus, hypertension, dyslipidemia, chronic kidney disease, medication use, and adrenal lesion characteristics, particularly lesion size. Covariates were selected for each multivariable model according to the specific outcome being evaluated rather than using one identical set of variables for all outcomes. Age and sex were retained as a priori demographic covariates. Other variables were considered based on clinical relevance, previously reported associations, potential confounding relationships, and their availability in the study dataset.

For models evaluating obesity, variables directly defining obesity were not entered simultaneously as independent covariates. Similarly, BMI and waist circumference were not simultaneously included where their inclusion produced clinically meaningful multicollinearity. For models evaluating diabetes, hypertension, dyslipidemia, and metabolic syndrome, covariates were selected according to their potential confounding role and the specific outcome definition. Adrenal lesion size and relevant radiological characteristics were considered in models evaluating factors associated with MACS. This outcome-specific approach was used to reduce inappropriate adjustment for variables that were components or potential intermediates of the outcome under investigation.

Statistical analysis

Data were entered into a computerized database and analyzed using SPSS Statistics version 27.0 (IBM Corp., Armonk, NY, USA). Data cleaning was performed before the final analysis. Continuous variables were assessed for distribution using appropriate graphical and statistical methods. Normally distributed continuous variables were presented as mean ± standard deviation, whereas skewed variables were presented as median with interquartile range. Categorical variables were summarized as frequencies and percentages.

The prevalence of MACS was calculated as the number of participants fulfilling the predefined MACS criteria divided by the total number of participants with valid and complete MACS assessment and expressed as a percentage with a 95% confidence interval (CI). The MACS and NFAI groups were compared with respect to demographic, clinical, anthropometric, biochemical, hormonal, and radiological characteristics. The independent-samples t-test was used for normally distributed continuous variables, whereas the Mann-Whitney U test was used for non-normally distributed continuous variables. Pearson’s chi-square test was used for categorical variables, while Fisher’s exact test was applied when expected cell counts were small.

Binary logistic regression was used to evaluate associations between MACS and individual metabolic outcomes, including obesity, hypertension, diabetes mellitus, dyslipidemia, metabolic syndrome, and the presence of multiple metabolic abnormalities. Crude odds ratios (ORs) and adjusted odds ratios (aORs) were reported with 95% CIs. Separate outcome-specific multivariable models were constructed, and the covariates included in each final model were selected according to the clinical relevance of the outcome, potential confounding, and prespecified study variables. This approach was used rather than describing the adjustment set as a single broad group of “clinically relevant confounders.”

Multicollinearity among covariates was assessed before construction of the final multivariable models. Model fit and influential observations were assessed using appropriate diagnostic procedures. A two-sided p-value <0.05 was considered statistically significant. Interpretation emphasized the magnitude and precision of effect estimates, including aORs and their 95% CIs, rather than statistical significance alone.

Missing data management

The study used prospective data collection and immediate review of completed forms to minimize missing information. Each questionnaire was checked for completeness before the participant’s data were finalized. Missing values were verified against medical records, laboratory reports, and radiological reports whenever possible. Participants with missing essential information required for classification of MACS, particularly post-DST cortisol, ACTH, or DHEA-S, were not considered to have a valid complete endocrine assessment and were excluded from the primary prevalence denominator if their cortisol autonomy could not be reliably classified. The number and reasons for such exclusions were documented.

Quality control

A standardized study protocol and data collection manual were prepared before recruitment. Research personnel were trained in participant recruitment, informed consent procedures, questionnaire administration, anthropometric measurements, blood pressure measurement, clinical assessment, specimen collection, data recording, and confidentiality. Anthropometric equipment was calibrated according to institutional procedures. Blood pressure equipment was checked before use. Laboratory investigations were performed using standardized laboratory procedures at Allama Iqbal Medical College, Lahore, with established internal quality control systems. The timing of dexamethasone administration and cortisol sampling was recorded for each participant. Completed forms were reviewed by the study investigators. A proportion of records was independently checked against original clinical, laboratory, and radiological records to identify transcription and classification errors.

Questionnaire development and pilot testing

The data collection questionnaire was developed according to the study objectives and incorporated demographic, lifestyle, medical, medication, clinical, anthropometric, blood pressure, radiological, biochemical, hormonal, and metabolic variables. Before formal recruitment, the questionnaire underwent face and content review by experts with relevant expertise in endocrinology, internal medicine, radiology, laboratory medicine, epidemiology, and research methodology.

The questionnaire was pilot tested among 30 participants with characteristics similar to the intended study population. Pilot participants were not included in the final analytical sample. The pilot phase assessed the clarity of questions, feasibility of participant interviews, average completion time, availability of clinical and laboratory information, completeness of responses, understanding of response categories, and practical feasibility of the endocrine testing procedure. Particular attention was given to accurate recording of dexamethasone administration time, cortisol collection time, ACTH, DHEA-S, medication use, and laboratory results. Any ambiguity, duplication, or difficulty identified during pilot testing was corrected before initiation of the main study. The final version of the questionnaire was then used consistently throughout the main study.

Standardization and reliability

Research personnel were trained before the beginning of formal data collection. Standardized instructions were used for anthropometric measurements, blood pressure measurement, clinical assessment, and questionnaire administration. Where feasible during pilot testing, duplicate anthropometric and blood pressure measurements were obtained to assess consistency between observers. Differences between observers were discussed and corrected through additional training.

Laboratory-derived variables were extracted directly from official laboratory reports. Radiological measurements were obtained from formal radiology reports and, where feasible, review of the original images. Hormonal measurements were recorded using the actual laboratory values rather than investigator-estimated categories.

Minimization of bias

Selection bias was minimized through consecutive recruitment of all eligible participants during the study period. Recruitment was not restricted according to age, sex, obesity, diabetes, hypertension, or cortisol status. Measurement bias was minimized through standardized procedures, calibrated equipment, predefined operational definitions, standardized laboratory assessment, and consistent hormonal testing. Because the study was conducted at a single tertiary-care hospital, the possibility of hospital-based selection and referral bias was acknowledged. The cross-sectional design also limits assessment of temporal relationships between MACS and metabolic abnormalities. Residual confounding from unmeasured or incompletely measured factors could not be completely excluded.

Information bias was reduced by obtaining information from medical records and laboratory reports wherever possible rather than relying solely on participant recall. Medication histories were verified against available prescriptions or medical records when feasible. Potential confounding was addressed through prospective measurement of relevant variables and outcome-specific multivariable regression analysis.

Ethical considerations

The Ethical Committee, Allama Iqbal Medical College, issued approval for this study (reference number: 213/DE/AIMC; dated: 9/7/2024). Written informed consent was obtained from all participants after the study procedures, objectives, potential risks, benefits, confidentiality arrangements, and voluntary nature of participation had been explained. Participation was voluntary, and participants were informed that they could withdraw from the study without affecting their clinical care. The study did not interfere with clinically indicated treatment. Participants with clinically important abnormalities, including newly identified diabetes, severe hypertension, significant cortisol excess, suspected pheochromocytoma, or radiological findings suggestive of malignancy, were referred to the relevant clinical team for appropriate evaluation and management. All study information was kept confidential. Unique identification numbers were used instead of participant names in the research database. Electronic data were password-protected and were accessible only to authorized members of the research team.

## Results

Among the 380 patients with adrenal incidentaloma, 95 (25.0%) had MACS, while 285 (75.0%) had NFAI. Patients with MACS were slightly older and had greater sedentary time than those without MACS. Clinically important differences were observed for hypertension, present in 64 (67.4%) patients with MACS compared with 154 (54.0%) without MACS (p = 0.019), and diabetes mellitus, present in 35 (36.8%) versus 71 (24.9%), respectively (p = 0.021). Dyslipidemia was also more frequent among patients with MACS (45 (47.4%) vs. 99 (34.7%), p = 0.028). Hypertension and diabetes had longer durations in the MACS group, while antihypertensive and antidiabetic medication use was also significantly more common. No significant differences were observed in sex, residence, educational status, smoking, or family history of diabetes or hypertension (Table 1).

**Table 1:** Sociodemographic, lifestyle, clinical, and medication characteristics according to MACS status. A p-value <0.05 was considered statistically significant. MACS = mild autonomous cortisol secretion

Variable		Total (N = 380)	MACS (n = 95)	Non-functioning adrenal incidentaloma (n = 285)	Statistical test	Test statistic	P-value
Age, years	Mean ± SD	56.4 ± 9.8	58.2 ± 9.1	55.8 ± 10.0	Independent t-test	t = 2.17	0.042
Male sex	n (%)	152 (40.0)	32 (33.7)	120 (42.1)	χ² test	χ² = 2.11	0.138
Female sex	n (%)	228 (60.0)	63 (66.3)	165 (57.9)			
Urban residence	n (%)	110 (28.9)	24 (25.3)	86 (30.2)	χ² test	χ² = 0.84	0.362
Rural residence	n (%)	270 (71.1)	71 (74.7)	199 (69.8)			
Education level, n (%)	No formal education	142 (37.4)	39 (41.1)	103 (36.1)	χ² test	χ² = 1.75	0.215
	Primary	89 (23.4)	24 (25.3)	65 (22.8)			
	Secondary	95 (25.0)	20 (21.1)	75 (26.3)			
	Intermediate	38 (10.0)	8 (8.4)	30 (10.5)			
	Graduate/postgraduate	16 (4.2)	4 (4.2)	12 (4.2)			
Smoking status, n (%)	Current smoker	38 (10.0)	12 (12.6)	26 (9.1)	χ² test	χ² = 2.26	0.318
	Former smoker	27 (7.1)	9 (9.5)	18 (6.3)			
	Never smoker	315 (82.9)	74 (77.9)	241 (84.6)			
Physical activity, n (%)	Low	228 (60.0)	63 (66.3)	165 (57.9)	χ² test	χ² = 2.26	0.089
	Moderate	95 (25.0)	19 (20.0)	76 (26.7)			
	High	57 (15.0)	13 (13.7)	44 (15.4)			
Sedentary time, hours/day		6.8 ± 2.4	7.2 ± 2.3	6.6 ± 2.4	Independent t-test	t = 2.18	0.038
Family history of diabetes, n (%)		98 (25.8)	29 (30.5)	69 (24.2)	χ² test	χ² = 1.49	0.208
Family history of hypertension, n (%)		133 (35.0)	38 (40.0)	95 (33.3)	χ² test	χ² = 1.39	0.241
Hypertension, n (%)		218 (57.4)	64 (67.4)	154 (54.0)	χ² test	χ² = 5.18	0.019
Duration of hypertension, years		8.2 ± 6.5	9.8 ± 6.8	7.7 ± 6.3	Independent t-test	t = 2.12	0.012
Diabetes mellitus, n (%)		106 (27.9)	35 (36.8)	71 (24.9)	χ² test	χ² = 5.04	0.021
Duration of diabetes, years		6.4 ± 5.2	7.8 ± 5.6	5.7 ± 4.9	Independent t-test	t = 1.89	0.008
Dyslipidemia, n (%)		144 (37.9)	45 (47.4)	99 (34.7)	χ² test	χ² = 4.83	0.028
Cardiovascular disease, n (%)		42 (11.1)	15 (15.8)	27 (9.5)	χ² test	χ² = 2.89	0.102
Chronic kidney disease, n (%)		34 (8.9)	12 (12.6)	22 (7.7)	χ² test	χ² = 2.11	0.148
Osteoporosis, n (%)		19 (5.0)	8 (8.4)	11 (3.9)	Fisher’s exact	—	0.102
Current glucocorticoid use, n (%)		8 (2.1)	3 (3.2)	5 (1.8)	Fisher’s exact	—	0.412
Antihypertensive use, n (%)		198 (52.1)	58 (61.1)	140 (49.1)	χ² test	χ² = 4.06	0.038
Antidiabetic medication, n (%)		91 (23.9)	32 (33.7)	59 (20.7)	χ² test	χ² = 6.59	0.012
Lipid-lowering medication, n (%)		76 (20.0)	24 (25.3)	52 (18.2)	χ² test	χ² = 2.19	0.142

Patients with MACS showed a generally less favorable anthropometric and metabolic profile than those with NFAI (Table 2). Obesity was present in 34 (35.8%) patients with MACS compared with 64 (22.5%) without MACS (p = 0.011), while central obesity affected 58 (61.1%) versus 128 (44.9%) patients (p = 0.006), respectively. Blood pressure and glycemic and lipid parameters were also significantly less favorable in the MACS group. Radiologically, bilateral adrenal lesions were more frequent among MACS patients (24 (25.3%) vs. 45 (15.8%), p = 0.042), and MACS was associated with larger adrenal lesions. Hormonal assessment showed substantially higher post-DST cortisol and greater ACTH suppression in patients with MACS, with 42 (44.2%) MACS patients having low/suppressed ACTH compared with 34 (11.9%) in the non-functioning group (p < 0.001). Low DHEA-S was also more frequent among patients with MACS (28 (29.5%) vs. 40 (14.0%), p = 0.001).

**Table 2:** Anthropometric, blood pressure, biochemical, radiological, and hormonal characteristics according to MACS status. A p-value <0.05 was considered statistically significant. MACS = mild autonomous cortisol secretion; BMI = body mass index; BP = blood pressure; LDL-C = low-density lipoprotein cholesterol; HDL-C = high-density lipoprotein cholesterol; eGFR = estimated glomerular filtration rate; DST = dexamethasone suppression test; DHEA-S = dehydroepiandrosterone sulfate; ACTH = adrenocorticotropic hormone

Variable	Total (N = 380)	MACS (n = 95)	Non-functioning adrenal incidentaloma (n = 285)	Statistical test	Test statistic	P-value
Weight, kg	72.5 ± 12.8	76.3 ± 13.2	71.2 ± 12.4	Independent t-test	t = 3.31	0.001
Height, cm	162.4 ± 8.6	161.8 ± 8.4	162.6 ± 8.7	Independent t-test	t = -0.80	0.452
BMI, kg/m²	27.5 ± 4.6	29.1 ± 4.8	27.0 ± 4.4	Independent t-test	t = 3.77	<0.001
Obesity, n (%)	98 (25.8)	34 (35.8)	64 (22.5)	Pearson χ² test	χ² = 6.62	0.011
Waist circumference, cm	94.2 ± 11.5	98.6 ± 11.8	92.7 ± 11.0	Independent t-test	t = 4.29	<0.001
Central obesity, n (%)	186 (48.9)	58 (61.1)	128 (44.9)	Pearson χ² test	χ² = 7.43	0.006
Waist-to-hip ratio	0.92 ± 0.08	0.95 ± 0.07	0.91 ± 0.08	Independent t-test	t = 4.65	<0.001
Systolic BP, mmHg	138.5 ± 18.2	144.2 ± 17.8	136.6 ± 18.0	Independent t-test	t = 3.59	0.001
Diastolic BP, mmHg	86.4 ± 10.8	89.8 ± 10.5	85.3 ± 10.7	Independent t-test	t = 3.60	0.001
Fasting plasma glucose, mg/dL	108.5 ± 32.4	118.2 ± 35.6	105.3 ± 30.2	Independent t-test	t = 3.17	0.002
HbA1c, %	6.2 ± 1.4	6.6 ± 1.5	6.1 ± 1.3	Independent t-test	t = 2.91	0.003
Total cholesterol, mg/dL	198.4 ± 38.6	208.5 ± 40.2	195.0 ± 37.1	Independent t-test	t = 2.89	0.004
LDL-C, mg/dL	124.5 ± 34.2	134.8 ± 36.5	121.1 ± 32.8	Independent t-test	t = 3.25	0.001
HDL-C, mg/dL	42.8 ± 10.5	40.2 ± 9.8	43.6 ± 10.6	Independent t-test	t = -2.87	0.008
Triglycerides, mg/dL	158.6 ± 72.4	178.5 ± 78.2	152.0 ± 69.5	Independent t-test	t = 2.94	0.003
Serum creatinine, mg/dL	0.92 ± 0.28	0.95 ± 0.30	0.91 ± 0.27	Independent t-test	t = 1.15	0.218
eGFR, mL/min/1.73 m²	82.5 ± 18.4	80.2 ± 19.1	83.2 ± 18.1	Independent t-test	t = -1.34	0.168
CT n (%)	285 (75.0)	74 (77.9)	211 (74.0)	Pearson χ² test	χ² = 0.59	0.452
MRI n (%)	62 (16.3)	14 (14.7)	48 (16.8)			
Ultrasound	33 (8.7)	7 (7.4)	26 (9.1)			
Right adrenal lesion, n (%)	125 (32.9)	28 (29.5)	97 (34.0)	Pearson χ² test	χ² = 0.67	0.418
Left adrenal lesion, n (%)	186 (48.9)	43 (45.3)	143 (50.2)	Pearson χ² test	χ² = 0.69	0.412
Bilateral lesions, n (%)	69 (18.2)	24 (25.3)	45 (15.8)	Fisher’s exact test	—	0.042
Maximum lesion diameter, cm	2.8 ± 1.2	3.4 ± 1.3	2.6 ± 1.1	Independent t-test	t = 5.39	<0.001
Unenhanced CT attenuation, HU	18.5 ± 12.4	22.8 ± 14.2	17.1 ± 11.5	Independent t-test	t = 3.54	0.001
Homogeneous lesion, n (%)	304 (80.0)	71 (74.7)	233 (81.8)	Pearson χ² test	χ² = 2.19	0.128
Calcification, n (%)	23 (6.1)	8 (8.4)	15 (5.3)	Fisher’s exact test	—	0.298
Necrosis, n (%)	8 (2.1)	4 (4.2)	4 (1.4)	Fisher’s exact test	—	0.142
Hemorrhage, n (%)	5 (1.3)	2 (2.1)	3 (1.1)	Fisher’s exact test	—	0.482
Post-DST cortisol, nmol/L	68.5 ± 82.4	128.5 ± 95.2	48.5 ± 62.4	Independent t-test	t = 7.66	<0.001
Morning ACTH, pg/mL	16.8 ± 10.2	12.5 ± 8.4	18.2 ± 10.5	Independent t-test	t = -5.36	<0.001
DHEA-S, μg/dL	85.4 ± 48.6	72.5 ± 42.8	89.7 ± 49.8	Independent t-test	t = -3.25	0.004
Suppressed/low ACTH, n (%)	76 (20.0)	42 (44.2)	34 (11.9)	Pearson χ² test	χ² = 46.40	<0.001
Low DHEA-S, n (%)	68 (17.9)	28 (29.5)	40 (14.0)	Pearson χ² test	χ² = 11.56	0.001
Repeat DST performed, n (%)	95 (25.0)	38 (40.0)	57 (20.0)	Fisher’s exact test	—	<0.001

Metabolic abnormalities were significantly more prevalent among patients with MACS. In particular, metabolic syndrome was identified in 54 (56.8%) patients with MACS compared with 114 (40.0%) patients with NFAI (p = 0.005). Similarly, diabetes mellitus, dyslipidemia, hypertriglyceridemia, low HDL-C, and high LDL-C were significantly more frequent in the MACS group. The burden of multiple metabolic abnormalities was particularly notable, with 62 (65.3%) MACS patients having at least two metabolic abnormalities compared with 136 (47.7%) non-MACS patients (p = 0.003), while 42 (44.2%) versus 72 (25.3%) had at least three abnormalities (p = 0.001). Overall, 76 (80.0%) MACS patients had at least one metabolic abnormality compared with 190 (66.7%) patients without MACS (p = 0.012). No significant differences were observed for prediabetes, cardiovascular disease, or chronic kidney disease (Table 3).

**Table 3:** Prevalence of metabolic abnormalities according to MACS status. Categorical variables were compared using Pearson’s chi-square test or Fisher’s exact test, as appropriate. The number of metabolic abnormalities was compared using an independent-samples t-test. MACS = mild autonomous cortisol secretion; LDL-C = low-density lipoprotein cholesterol; HDL-C = high-density lipoprotein cholesterol

Metabolic outcome	Total (N = 380)	MACS (n = 95)	Non-functioning adrenal incidentaloma (n = 285)	Test statistic	P-value
Obesity, n (%)	98 (25.8)	34 (35.8)	64 (22.5)	χ² = 6.62	0.011
Central obesity, n (%)	186 (48.9)	58 (61.1)	128 (44.9)	χ² = 7.43	0.006
Hypertension, n (%)	218 (57.4)	64 (67.4)	154 (54.0)	χ² = 5.18	0.019
Diabetes mellitus, n (%)	106 (27.9)	35 (36.8)	71 (24.9)	χ² = 5.04	0.021
Prediabetes, n (%)	82 (21.6)	26 (27.4)	56 (19.6)	χ² = 2.51	0.112
Dyslipidemia, n (%)	144 (37.9)	45 (47.4)	99 (34.7)	χ² = 4.83	0.028
High triglycerides, n (%)	128 (33.7)	42 (44.2)	86 (30.2)	χ² = 6.28	0.014
Low HDL-C, n (%)	152 (40.0)	48 (50.5)	104 (36.5)	χ² = 5.85	0.016
High LDL-C, n (%)	114 (30.0)	38 (40.0)	76 (26.7)	χ² = 6.03	0.018
Metabolic syndrome, n (%)	168 (44.2)	54 (56.8)	114 (40.0)	χ² = 8.19	0.005
Cardiovascular disease, n (%)	42 (11.1)	15 (15.8)	27 (9.5)	χ² = 2.89	0.102
Chronic kidney disease, n (%)	34 (8.9)	12 (12.6)	22 (7.7)	χ² = 2.11	0.148
≥1 metabolic abnormality, n (%)	266 (70.0)	76 (80.0)	190 (66.7)	χ² = 6.03	0.012
≥2 metabolic abnormalities, n (%)	198 (52.1)	62 (65.3)	136 (47.7)	χ² = 8.79	0.003
≥3 metabolic abnormalities, n (%)	114 (30.0)	42 (44.2)	72 (25.3)	χ² = 12.18	0.001
Number of metabolic abnormalities	2.4 ± 1.6	3.1 ± 1.5	2.1 ± 1.5	t = 5.63	<0.001

After adjustment for relevant demographic, lifestyle, clinical, radiological, and other prespecified confounders, obesity and adrenal lesion size remained independently associated with MACS. Obese patients had significantly higher odds of MACS (aOR = 1.78, 95% CI = 1.04-3.05, p = 0.036), while each 1-cm increase in adrenal lesion size was associated with higher odds of MACS (aOR = 1.38, 95% CI = 1.14-1.67, p = 0.001). In the outcome-specific models, MACS remained significantly associated with obesity in 34 (35.8%) patients (aOR = 1.85, 95% CI = 1.09-3.14, p = 0.022), diabetes mellitus in 35 (36.8%) patients (aOR = 1.68, 95% CI = 1.01-2.80, p = 0.046), metabolic syndrome in 54 (56.8%) patients (aOR = 1.82, 95% CI = 1.11-2.98, p = 0.017), and the presence of at least two metabolic abnormalities in 62 (65.3%) patients (aOR = 1.92, 95% CI = 1.17-3.15, p = 0.010) (Table 4).

**Table 4:** Multivariable logistic regression analysis of factors associated with MACS and metabolic abnormalities. Binary logistic regression was used to estimate crude and adjusted ORs with 95% CIs. The MACS model was adjusted for age, sex, obesity, hypertension, diabetes mellitus, dyslipidemia, smoking status, physical activity, bilateral adrenal lesions, and lesion size. The outcome-specific models for obesity, hypertension, diabetes mellitus, dyslipidemia, metabolic syndrome, and ≥2 metabolic abnormalities were adjusted for MACS and the prespecified demographic, lifestyle, clinical, medication, renal function, and adrenal lesion covariates relevant to each outcome. The exact covariates included in each adjusted model are indicated in the table. Variables constituting the respective outcome were not simultaneously entered into the corresponding model to minimize overadjustment and collinearity. A two-sided p-value <0.05 was considered statistically significant. MACS = mild autonomous cortisol secretion; OR = odds ratio; CI = confidence interval

Outcome/Predictor		Crude OR	95% CI	P-value	Adjusted OR	95% CI	P-value	Covariates included in adjusted model
Outcome: MACS	Age, per year	1.03	1.00–1.06	0.045	1.02	0.99–1.05	0.128	Age, sex, obesity, hypertension, diabetes mellitus, dyslipidemia, smoking status, physical activity, bilateral adrenal lesion, lesion size
	Male sex	0.70	0.43–1.14	0.152	0.68	0.41–1.13	0.138	
	Obesity	1.92	1.15–3.21	0.012	1.78	1.04–3.05	0.036	
	Hypertension	1.76	1.08–2.87	0.023	1.58	0.94–2.65	0.082	
	Diabetes mellitus	1.76	1.08–2.88	0.023	1.52	0.90–2.56	0.118	
	Dyslipidemia	1.71	1.08–2.71	0.022	1.48	0.91–2.41	0.112	
	Smoking	1.44	0.72–2.88	0.302	1.38	0.67–2.84	0.382	
	Low physical activity	1.45	0.88–2.38	0.142	1.32	0.79–2.21	0.288	
	Bilateral adrenal lesion	1.82	1.04–3.18	0.036	1.75	0.98–3.12	0.058	
	Lesion size, per cm	1.42	1.18–1.71	<0.001	1.38	1.14–1.67	0.001	
Outcome: Obesity	MACS	1.92	1.15–3.21	0.012	1.85	1.09–3.14	0.022	MACS, age, sex, smoking status, physical activity, hypertension, diabetes mellitus, dyslipidemia, renal function, adrenal lesion characteristics
Outcome: Hypertension	MACS	1.76	1.08–2.87	0.023	1.62	0.98–2.68	0.058	
Outcome: Diabetes mellitus	MACS	1.76	1.08–2.88	0.023	1.68	1.01–2.80	0.046	
Outcome: Dyslipidemia	MACS	1.71	1.08–2.71	0.022	1.58	0.98–2.55	0.060	
Outcome: Metabolic syndrome	MACS	1.96	1.22–3.15	0.006	1.82	1.11–2.98	0.017	
Outcome: ≥2 metabolic abnormalities	MACS	2.04	1.27–3.28	0.003	1.92	1.17–3.15	0.010	

## Discussion

The present study identified MACS in 95 of 380 patients with adrenal incidentaloma (25.0%), indicating that one in four patients had biochemical evidence of MACS. This frequency was consistent with the prevalence reported internationally, where MACS has generally been identified in approximately 20-50% of patients with adrenal adenomas, although estimates vary according to the diagnostic criteria, population characteristics, and intensity of hormonal assessment [1]. Earlier studies using the terminology of “subclinical Cushing syndrome” reported lower frequencies, illustrating how changes in diagnostic definitions and testing strategies influence reported prevalence [14]. The observed prevalence in the current study therefore supports the growing recognition that autonomous cortisol secretion is relatively common among apparently asymptomatic patients with adrenal incidentaloma. Importantly, Pakistani evidence specifically addressing MACS remains limited; available Pakistani literature has largely consisted of case reports or descriptive reports of adrenal incidentalomas rather than systematically evaluating MACS and its metabolic consequences. A review emphasized the need for biochemical assessment of adrenal incidentalomas, including evaluation for cortisol excess, while a Pakistani case report similarly highlighted the importance of appropriate long-term assessment of adrenal incidentalomas [15,16].

The demographic findings showed that patients with MACS were modestly older than those with NFAI, while sex distribution did not differ significantly. The predominance of patients in the sixth decade is broadly compatible with previous adrenal incidentaloma cohorts, in which detection is particularly common among middle-aged and older adults. Kim et al. reported the highest frequency of adrenal incidentalomas in the sixth and seventh decades of life [17]. The lack of a significant sex difference in MACS in the present study is also noteworthy because adrenal incidentalomas overall have been reported in both sexes, with some cohorts showing a modest female predominance. The present findings suggest that, within this Pakistani population, age rather than sex may be more relevant to the identification of MACS, although the relatively modest difference in age indicates that age alone is unlikely to explain the observed metabolic phenotype.

A particularly important finding was the greater burden of cardiometabolic abnormalities among patients with MACS. Hypertension, diabetes mellitus, and dyslipidemia were more frequent in the MACS group than in patients with NFAI. These findings are consistent with international evidence, including a systematic review and meta-analysis showing increased risks of diabetes, hypertension, and dyslipidemia among patients with MACS [5]. Similar differences have also been reported in contemporary cohorts of patients with adrenal incidentaloma [18]. The longer duration of hypertension and diabetes observed among patients with MACS may further indicate a greater cumulative cardiometabolic burden, although the cross-sectional design prevents establishing whether MACS preceded these conditions.

The anthropometric findings further supported an adverse metabolic profile in MACS. Patients with MACS had higher BMI, waist circumference, and waist-to-hip ratio, with central obesity being particularly prominent. They also demonstrated less favorable blood pressure, glycemic, and lipid parameters. This pattern is biologically plausible, as chronic mild cortisol excess may be associated with visceral adiposity, insulin resistance, hypertension, and adverse lipid metabolism. Previous reviews have similarly linked MACS with obesity, impaired glucose metabolism, dyslipidemia, cardiovascular risk, and other metabolic complications [1,19]. Recent metabolic profiling has also suggested a continuum between non-functioning adrenal tumors, MACS, and overt adrenal Cushing syndrome, with progressively greater metabolic disturbances accompanying increasing cortisol exposure [20]. The predominance of central adiposity in the present study is clinically relevant because visceral fat is strongly associated with insulin resistance and cardiovascular risk.

The hormonal findings were consistent with the expected biochemical phenotype of MACS. Patients with MACS had higher post-dexamethasone cortisol and lower morning ACTH and DHEA-S concentrations than those with NFAI. A greater proportion also demonstrated low or suppressed ACTH, supporting the interpretation of predominantly ACTH-independent cortisol secretion. Previous studies have reported similar hormonal patterns in patients with adrenal incidentaloma and subclinical cortisol excess [14]. Current international guidance considers the 1-mg overnight DST central to the assessment of MACS, with evaluation of ACTH independence and associated comorbidities also recommended [3]. The concordance of post-DST cortisol with reduced ACTH and DHEA-S in the present study therefore provides additional biochemical support for the classification of MACS.

The radiological findings further suggested a relationship between adrenal morphology and cortisol autonomy. Patients with MACS had larger adrenal lesions, with a mean maximum diameter of 3.4 ± 1.3 cm, compared with 2.6 ± 1.1 cm in the non-functioning group, and bilateral lesions were more frequent in MACS patients (24 (25.3%) vs. 45 (15.8%)). Importantly, lesion size remained independently associated with MACS after adjustment, with each 1-cm increase in lesion diameter associated with a 38% increase in the odds of MACS (aOR = 1.38, 95% CI = 1.14-1.67). This finding is clinically relevant because adrenal lesion size is routinely assessed during incidentaloma evaluation, although size alone cannot establish hormonal functionality. Previous literature has demonstrated that most adrenal incidentalomas are relatively small and benign, while autonomous cortisol secretion occurs across a range of lesion sizes [17,21]. The association observed here therefore suggests that larger lesions may warrant particularly careful hormonal evaluation, rather than implying that larger size is diagnostic of MACS.

The strongest clinical finding was the substantially greater burden of multiple metabolic abnormalities among patients with MACS. Metabolic syndrome was present in 54 (56.8%) MACS patients compared with 114 (40.0%) patients without MACS, while 62 (65.3%) MACS patients had at least two metabolic abnormalities and 42 (44.2%) had at least three. The overall number of metabolic abnormalities was also significantly greater among MACS patients. These findings extend the individual associations with obesity, diabetes, hypertension, and dyslipidemia and suggest that MACS may contribute to a broader cardiometabolic phenotype rather than an isolated metabolic abnormality. This interpretation is consistent with the large meta-analysis demonstrating an increased prevalence of several cardiometabolic comorbidities in MACS and with contemporary reviews emphasizing that MACS is associated with increased cardiovascular risk and mortality [1,2,22]. Nevertheless, the present cross-sectional design means that the findings should be interpreted as associations rather than evidence that MACS caused these metabolic abnormalities.

The adjusted analyses strengthened several of these observations. Obesity remained independently associated with MACS (aOR = 1.78, 95% CI = 1.04-3.05), while larger adrenal lesion size remained an independent predictor (aOR = 1.38, 95% CI = 1.14-1.67). Furthermore, MACS remained independently associated with obesity, diabetes mellitus, metabolic syndrome, and the presence of at least two metabolic abnormalities after adjustment, with the strongest association observed for having two or more metabolic abnormalities (aOR = 1.92, 95% CI = 1.17-3.15). In contrast, the associations with hypertension and dyslipidemia were attenuated after adjustment, suggesting that some of their crude associations may have been explained by age, adiposity, medication use, lifestyle, renal function, or other clinical factors. This distinction is important because MACS commonly occurs in individuals who already have conventional cardiometabolic risk factors. However, the persistence of associations with diabetes and overall metabolic syndrome after multivariable adjustment supports the possibility that autonomous cortisol secretion contributes independently to metabolic deterioration. Similar evidence from international studies has shown a higher cardiometabolic burden in MACS, although the magnitude of associations varies considerably between populations and diagnostic thresholds [18,23].

Strengths

The major strengths of this study included its single-center design at Allama Iqbal Medical College, Lahore, which provided a well-defined clinical setting for comprehensive assessment of patients with adrenal incidentaloma. The study incorporated comprehensive clinical, anthropometric, biochemical, hormonal, and radiological assessment rather than relying solely on imaging or a single biochemical marker. MACS was evaluated using DST with additional assessment of ACTH and DHEA-S, allowing biochemical characterization of cortisol autonomy. The inclusion of a clearly defined NFAI comparison group was another important strength because it allowed the metabolic burden associated with MACS to be examined within the same clinical population. Furthermore, the study assessed multiple metabolic outcomes and used multivariable logistic regression to control for important potential confounders, including age, sex, adiposity, lifestyle factors, medication use, renal function, and adrenal characteristics. These features strengthened the internal validity of the observed associations and provided locally relevant evidence from a population for which published data on MACS remain limited.

Limitations

Several limitations should be considered when interpreting these findings. First, the cross-sectional design precludes establishing temporal relationships or causality between MACS and metabolic abnormalities; therefore, the observed associations should not be interpreted as evidence that MACS directly caused obesity, hypertension, diabetes mellitus, dyslipidemia, or metabolic syndrome. Second, the study was conducted at a single tertiary-care center at Allama Iqbal Medical College, Lahore, which may limit the generalizability of the findings to other regions of Pakistan and community-based populations. Although clinically relevant covariates were incorporated into the multivariable models, residual confounding from dietary patterns, socioeconomic status, genetic susceptibility, medication exposure, and unmeasured lifestyle factors cannot be excluded. Third, biochemical classification of MACS may be affected by biological and analytical variability in cortisol and related hormones, and repeated hormonal assessments were not available for all participants. The relatively small number of participants with less frequent outcomes, particularly cardiovascular disease and chronic kidney disease, may also have limited the statistical power to detect modest associations. Finally, because metabolic syndrome comprises several interrelated components, including obesity, hypertension, glucose abnormalities, and dyslipidemia, outcome-specific model specification was used to minimize overadjustment and multicollinearity. In addition, the large number of univariate comparisons performed in the descriptive analyses may have increased the possibility of type I error; consequently, these findings were interpreted cautiously, and the multivariable analyses were given greater emphasis.

## Conclusions

MACS was identified in a substantial proportion of patients with adrenal incidentaloma and was associated with an adverse cardiometabolic profile, particularly obesity, central adiposity, diabetes mellitus, dyslipidemia, metabolic syndrome, and clustering of multiple metabolic abnormalities. Several of these associations remained significant after adjustment for relevant demographic, lifestyle, clinical, medication-related, and adrenal lesion characteristics, supporting an independent association between MACS and metabolic risk. These findings underscore the importance of hormonal evaluation for MACS and systematic assessment of cardiometabolic comorbidities in patients with adrenal incidentaloma, particularly in Pakistan, where evidence remains limited. Prospective multicenter studies with longitudinal follow-up are needed to clarify the temporal and causal relationships between MACS and metabolic outcomes and to determine whether targeted treatment, including adrenalectomy or optimized medical management where appropriate, can reduce long-term cardiometabolic morbidity and mortality.

## Appendices

**Table 5:** Study data collection questionnaire.

Variable / Domain	Response / Sub-variable / Coding / Measurement
Participant identification	
Study identification number	
Recruitment date	
Recruitment department	Endocrinology / Medicine / Surgery / Urology / Radiology / Other
Source of referral	Outpatient / Inpatient / Emergency / Imaging department / Other
Reason for original imaging	Abdominal pain / Renal or urinary symptoms / Gastrointestinal symptoms / Trauma / Suspected malignancy / Other
Date adrenal lesion identified	
Previous adrenal evaluation	Yes / No
Sociodemographic characteristics	
Age	Completed years; continuous
Sex	Male / Female
Residence	Urban / Rural
District	
Marital status	Married / Unmarried / Widowed / Divorced
Education	No formal education / Primary / Middle / Secondary / Intermediate / Graduate / Postgraduate
Occupation	Government / Private / Self-employed / Farmer / Homemaker / Retired / Unemployed / Other
Monthly household income	PKR; continuous or predefined categories
Lifestyle characteristics	
Smoking status	Never / Former / Current
Smoking duration	Years
Cigarette consumption	Cigarettes/day
Pack-years	Calculated
Smokeless tobacco use	Never / Former / Current
Type of tobacco	Naswar / Chewing tobacco / Other
Physical activity	Low / Moderate / High
Moderate physical activity	Minutes/week
Vigorous physical activity	Minutes/week
Sedentary time	Hours/day
Dietary pattern	Home-prepared / Mixed / Predominantly processed or restaurant food
Fruit/vegetable intake	Servings/day
Sugar-sweetened beverages	Never / <1/week / 1–6/week / ≥1/day
Medical history	
Hypertension	Yes / No
Duration of hypertension	Years
Diabetes mellitus	Yes / No
Duration of diabetes	Years
Prediabetes	Yes / No / Unknown
Dyslipidemia	Yes / No
Cardiovascular disease	Yes / No
Coronary artery disease	Yes / No
Previous myocardial infarction	Yes / No
Heart failure	Yes / No
Cerebrovascular disease	Yes / No
Chronic kidney disease	Yes / No
Osteoporosis	Yes / No / Unknown
Previous fragility fracture	Yes / No
Liver disease	Yes / No
Thyroid disease	Yes / No
Previous endocrine disease	Yes / No
Family history of diabetes	Yes / No
Family history of hypertension	Yes / No
Family history of adrenal disease	Yes / No
Medication history	
Current medication use	Yes / No
Antihypertensive medication	Yes / No; name
Number of antihypertensive drugs	
Antidiabetic medication	Yes / No; name
Insulin use	Yes / No
Lipid-lowering medication	Yes / No
Statin use	Yes / No
Oral estrogen therapy	Yes / No / Not applicable
Oral contraceptive use	Yes / No / Not applicable
Antiepileptic medication	Yes / No; name
Rifampicin use	Yes / No
Systemic glucocorticoid use	Yes / No
Inhaled glucocorticoid use	Yes / No
Topical glucocorticoid use	Yes / No
Other relevant medication	Yes / No; specify
Clinical assessment	
Cushingoid appearance	Present / Absent
Facial plethora	Present / Absent
Easy bruising	Present / Absent
Proximal muscle weakness	Present / Absent
Wide violaceous striae	Present / Absent
Unexplained weight gain	Present / Absent
Osteoporosis/fracture	Present / Absent
Menstrual irregularity	Present / Absent / Not applicable
Hirsutism	Present / Absent / Not applicable
Clinical suspicion of overt Cushing syndrome	Yes / No
Anthropometric assessment	
Weight	kg; measured
Height	cm; measured
BMI	kg/m²; calculated
BMI category	Predefined category
Waist circumference	cm; measured
Hip circumference	cm; measured
Waist-to-hip ratio	Calculated
Central obesity	Yes / No
Blood pressure	
Systolic BP, reading 1	mmHg
Diastolic BP, reading 1	mmHg
Systolic BP, reading 2	mmHg
Diastolic BP, reading 2	mmHg
Systolic BP, reading 3	mmHg, if required
Diastolic BP, reading 3	mmHg, if required
Mean systolic BP	mmHg
Mean diastolic BP	mmHg
Hypertension status	Yes / No
Controlled hypertension	Yes / No
Adrenal imaging	
Imaging modality	CT / MRI / Ultrasound
Laterality	Right / Left / Bilateral
Number of lesions	One / Multiple
Maximum lesion diameter	cm
Right adrenal lesion size	cm
Left adrenal lesion size	cm
Morphology	Homogeneous / Heterogeneous
Unenhanced CT attenuation	HU
Contrast enhancement	Present / Absent
Absolute washout	%
Relative washout	%
Calcification	Present / Absent
Necrosis	Present / Absent
Hemorrhage	Present / Absent
Local invasion	Present / Absent
Lymphadenopathy	Present / Absent
Radiological diagnosis	Adenoma / Myelolipoma / Cyst / Pheochromocytoma suspected / Malignancy suspected / Indeterminate / Other
Biochemical assessment	
Fasting plasma glucose	mg/dL
HbA1c	%
Total cholesterol	mg/dL
LDL cholesterol	mg/dL
HDL cholesterol	mg/dL
Triglycerides	mg/dL
Serum creatinine	mg/dL
eGFR	mL/min/1.73 m²
Serum potassium	mmol/L
Serum sodium	mmol/L
Diabetes mellitus	Yes / No
Prediabetes	Yes / No
Dyslipidemia	Yes / No
Chronic kidney disease	Yes / No
Cortisol assessment	
1-mg overnight DST performed	Yes / No
Dexamethasone dose	1 mg
Dexamethasone administration date	DD/MM/YYYY
Dexamethasone administration time	HH:MM
Post-DST cortisol collection date	DD/MM/YYYY
Post-DST cortisol collection time	HH:MM
Post-DST serum cortisol	nmol/L
Post-DST cortisol	µg/dL where available
Dexamethasone concentration	Laboratory value, if measured
DST adequately performed	Yes / No
DST repeated	Yes / No
Repeat post-DST cortisol	nmol/L
ACTH and adrenal hormonal assessment	
Morning ACTH	pg/mL
ACTH interpretation	Suppressed / Low / Normal / Elevated
Morning DHEA-S	Laboratory value
DHEA-S interpretation	Low / Normal / High
24-hour urinary free cortisol	Laboratory value, if performed
Late-night salivary cortisol	Laboratory value, if performed
Plasma free metanephrines	Normal / Elevated / Not performed
Urinary fractionated metanephrines	Normal / Elevated / Not performed
Aldosterone	Laboratory value
Renin	Laboratory value
Aldosterone-renin ratio	Calculated
Hypokalemia	Yes / No
Evidence of primary aldosteronism	Yes / No / Not assessed
MACS classification	
Overt Cushing syndrome	Yes / No
Post-DST cortisol >50 nmol/L	Yes / No
ACTH independency	Confirmed / Not confirmed
Repeat DST required	Yes / No
Repeat DST abnormal	Yes / No
Final cortisol autonomy classification	MACS / Non-functioning adrenal incidentaloma / Overt Cushing syndrome / Other
Metabolic outcomes	
Obesity	Yes / No
Central obesity	Yes / No
Hypertension	Yes / No
Diabetes mellitus	Yes / No
Prediabetes	Yes / No
Dyslipidemia	Yes / No
Elevated triglycerides	Yes / No
Low HDL cholesterol	Yes / No
Elevated LDL cholesterol	Yes / No
Metabolic syndrome	Yes / No
Number of metabolic syndrome components	0–5
Cardiovascular disease	Yes / No
Final classification	
Study group	MACS / Non-functioning adrenal incidentaloma
Primary outcome	MACS present / MACS absent
Number of cardiometabolic abnormalities	Numeric
Included in final analysis	Yes / No
Reason for exclusion	Missing DST / Incomplete endocrine assessment / Overt Cushing syndrome / Other functional adrenal disorder / Malignancy / Other
Data verification completed	Yes / No
Investigator initials	
Date of final verification	
